# Total workflow uncertainty of frameless radiosurgery with the Gamma Knife Icon cone‐beam computed tomography

**DOI:** 10.1002/acm2.13564

**Published:** 2022-02-14

**Authors:** William N. Duggar, Bart Morris, Rui He, Claus Yang

**Affiliations:** ^1^ Department of Radiation Oncology University of MS Medical Center Jackson Mississippi USA

**Keywords:** CBCT, frameless, Gamma Knife Icon, stereotactic, uncertainty

## Abstract

**Objective:**

Frameless treatment with the Gamma Knife Icon is still relatively new as a treatment option. As a result, additional confidence/knowledge about the uncertainty that exists within each portion of the treatment workflow could be gained especially regarding steps that have not been previously studied in the literature.

**Methods:**

The Icon base delivery device (Perfexion) uncertainty is quantified and validated. The novel portions of the Icon such as mask immobilization, cone‐beam computed tomography image guidance, and the intrafraction motion management methods are studied specifically and to a greater extent to determine a total workflow uncertainty of frameless treatment with the Icon.

**Results:**

The uncertainty of each treatment workflow step has been identified with the total workflow uncertainty being identified in this work as 1.3 mm with a standard deviation of 0.51 mm.

**Conclusion:**

The total uncertainty of frameless treatment with the Icon has been evaluated and this data may indicate the need for setup margin in this setting with data that could be used by other institutions to calculate needed setup margin per their preferred recipe after validation of this data in their context.

## 1 INTRODUCTION

**FIGURE 1:**
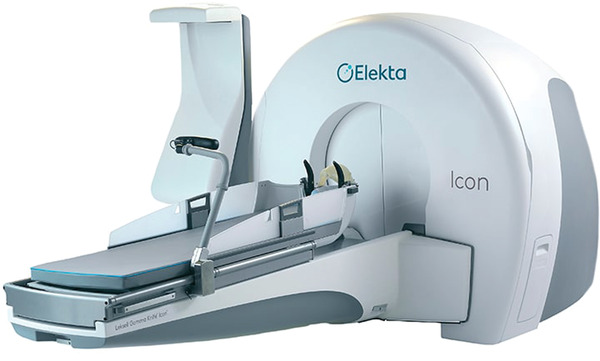
The Gamma Knife Icon with its base delivery system, mask immobilization, high‐definition motion management (HDMM), and cone‐beam computed tomography (CBCT) (Image courtesy of Elekta) © 2018 Elekta AB

The Leksell Gamma Knife has been used for decades in the treatment of various intracranial targets using stereotactic radiosurgery. Over the years, the design has changed several times as technology has evolved with the newest iteration being the Leksell Gamma Knife Icon (Elekta, Stockholm, Sweden), shown in Figure 1. The Icon (shortened name of the Gamma Knife Icon) utilizes pre‐treatment image guidance in the form of cone‐beam computed tomography (CBCT) and a high‐definition motion management (HDMM) camera which tracks infrared markers. The HDMM system is also known as the IFMM in certain Elekta manuals, software versions, and other literature, but this work will utilize the HDMM nomenclature. The treatment delivery mechanics match that of the previous generation known as the Perfexion aside from the already discussed additions. Whereas the Perfexion relies on an indicator box attached to the stereotactic frame during pre‐treatment magnetic resonant imaging (MRI) and/or CT imaging, the CBCT can now define the stereotactic coordinate system needed for treatment planning via the process of co‐registration of these images with the CBCT. Because of this image guidance technology, a less invasive immobilization of a thermoplastic mask can be molded to a patient's head and face instead of the traditional stereotactic frame. Though the masks are less invasive, they are less rigid than the frame. Since imaging only occurs prior to treatment and only again as needed when the patient is out of position, the patient's position must be monitored post‐CBCT to ensure that the patient remains in the same position from imaging to treatment completion. This monitoring is performed by the HDMM, which compares the position of a temporary fiducial placed on the patient's nose to four stationary, reference reflectors on the mask frame.^1^, ^2^

### 1.1 Frameless Gamma Knife Icon workflow

Patients can be treated in one single or multiple fractions utilizing the frameless immobilization technique. They first undergo high‐quality MRI imaging with minimal voxel size (i.e. 1 x 1 x 1 mm^3^) in the x, y, and z coordinate systems. These images are sent to the treatment planning system for Gamma Knife, GammaPlan, where an examination is created for that patient. With the latest GammaPlan versions, the MRI images (or CT if needed) are first used to determine the head surface for dose calculation and can be used for pre‐planning. Then, the targets are contoured on the MRI as well as any organs at risk near the treatment area. The target grid is set over the area surrounding the target volume and the prescription dose is defined. At this point, depending on the nature of the target and histology, a treatment plan is developed via forward or inverse planning to place the “shots” throughout the target volume resulting in an acceptable target coverage by the prescription dose and acceptable sparing of any nearby normal tissues including the surrounding healthy brain tissue. Each “shot” represents a couch position which leads to the radiation focal point being located at a prescribed intracranial position.

The treatment plan cannot be completed until the patient is simulated in treatment position on the Icon with the creation of a patient‐specific thermoplastic mask and head cushion combination for use with the Gamma Knife mask adaptor. Once the mask and head cushion are set (about 30 min in our experience to limit later mask shrinkage), CBCT imaging is acquired of the patient in treatment position and these images are also transferred to GammaPlan and used as a reference image set to define the initial stereotactic coordinate system. The pre‐planning images must be co‐registered with the CBCT. Stereotactic space is now defined over the patient's anatomy, creating a physical location for each “shot” and allowing clearance analysis of the patient within the Gamma Knife unit. Once planning is completed and any clearance issues are resolved, the plan is electronically approved, printed, and exported to the Icon for treatment delivery.

Before treatment delivery, the patient is again set up in treatment position using their now hardened mask and cushion with the addition of a fiducial marker placed on the nose tip, and a CBCT is repeated. This new CBCT is transferred to GammaPlan and co‐registered with the reference CBCT. The plan is then re‐calculated after “shot” position translation based on the co‐registration and the new dose distribution can be compared to the original plan. Note that the treatment plan is corrected to an updated stereotactic coordinate system with this workflow, whereas with linac treatment, the patient is corrected back to the initial position. Upon approval, the workflow returns to the treatment console for treatment delivery and the HDMM camera sets its baseline for tracking and from that moment, the fiducial marker is tracked to stay within a threshold distance in any direction set by the treatment provider (i.e., 1.5 mm) of its original position or else treatment is paused. If the fiducial goes beyond this threshold temporarily, treatment is paused, but if the fiducial remains beyond the threshold, the patient will be automatically pulled out of the machine until the CBCT imaging step can be repeated and a new baseline is set. This process continues until treatment delivery is completed. As can be seen, frameless Gamma Knife treatment contains many steps, each with its own potential uncertainty, just as with other methods of radiation treatment delivery.

### 1.2 Total workflow uncertainty

Uncertainty in radiation therapy may lead to issues with either precision or accuracy of treatment delivery. The management and mitigation of uncertainty is the primary aim of quality assurance and control. Professional guidelines from the American Association of Physicists in Medicine (AAPM) and others have identified known sources of error and recommended acceptable tolerances depending on the type of treatment to be delivered.^3^, ^4^, ^5^, ^6^, ^7^ Uncertainty exists at each step of the treatment process and may combine with much larger overall uncertainties. The Gamma Knife has a stellar reputation of treatment delivery with < 1 mm accuracy with only a few detractors.^8^, ^9^ Though the accuracy of the Gamma Knife may be commendable, the end‐to‐end accuracy, including all workflow steps, is perhaps more clinically relevant than that of any portion. For this reason, end‐to‐end tests can be important and quite useful such as two groups did with polymer gels.^10^, ^11^ Such tests are also endorsed by the recent AAPM Task Group (TG) on Gamma Knife treatment.^7^

Alternatively, the uncertainty within each workflow step could be quantified, which is demonstrated during the commissioning of a new treatment device.^2^ Two examples are a measurement of the traditional G‐frame uncertainty to be on the order of < 0.5 mm and that of the patient positioning system to be < 0.25 mm.^12^, ^13^ Other authors have confirmed these results leading to well‐defined confidence in the base delivery component of the Icon.^14^, ^15^ Previous studies have indicated MRI distortion as a major source of error in Gamma Knife treatment as uncertainties have been reported close to 1 mm, though methods to improve distortion have been described.^16^, ^17^

With less invasive and less rigid immobilization, frameless radiosurgery/therapy potentially means more uncertainty, both during treatment (intra‐fractional) and between treatment sessions (inter‐fractional). The beam‐specific treatment depth for each shot may change due to variability in both patient setup within the mask and movement during treatment. Also, residual error may exist despite the co‐registration correction with the pre‐treatment CBCT.^18^, ^19^, ^20^, ^21^ Tumors themselves have been found to change in shape, size, or position over time.^22^ The convenience of the frameless workflow forces a tradeoff with stability and potentially total accuracy of the delivered treatment. In the design of the Icon, the CBCT and HDMM are meant to mitigate this inter‐ and intra‐fractional error, but the extent of this mitigation has not been studied as comprehensively as the traditional frame‐based method, especially considering the fact that frameless treatment with the Icon may happen over multiple fractions rather than only in a single treatment session.

### 1.3 Uncertainty and frameless radiosurgery

A radiosurgery method should demonstrate high levels of both accuracy and precision throughout its clinical use.^23^ Tumor control probability and arteriovenous malformation control have been shown to suffer when the accuracy level goes beyond 1 mm.^24^ Others have investigated this frameless workflow to an extent, but key workflow steps such as intrafraction motion were admittedly left out.^2^, ^25^ This work seeks to evaluate quantitatively the uncertainty arising from each critical step of the frameless Gamma Knife workflow and compare it to the traditionally held belief of sub‐mm accuracy for the traditional frame‐based method.^23^ Specific attention is given to the immobilization, image guidance, and motion management methods as they are novel to the frameless Gamma Knife process. Each source of uncertainty measured in this work is designated as “systematic” meaning that every patient experiences the uncertainty to the same extent or “random” meaning that the application of that uncertainty to each patient varies. This terminology is consistent with clinical margin theory that has been traditionally utilized for the application of setup margin to target volumes in radiation therapy.^26^, ^27^, ^28^, ^29^ Systematic error will result in a geometric “shift” of the radiation dose distribution whereas random uncertainty will be more of a “blurring” effect. Categorization and inclusion of both systematic and random components into setup margin estimation are essential to an adequate and rational setup margin.^30^ Additionally, uncertainty can be classified as Type A or Type B based on whether it is the result of repeated, direct measurements or whether it is secondary from a manufacturer or published literature, respectively.^31^ MRI distortion is the only example in this work of Type B uncertainty. A summary of the targeted steps for uncertainty evaluation can be seen in Table 1.

**TABLE 1:** Types of uncertainty to be considered in the Gamma Knife workflow and the intended sources for measurement of each

**Uncertainty**	**Description**	**Measurement source**	**Uncertainty type**
MRI distortion	Distortion of MRI images	Phantom Tests	Systematic
Couch position	Accuracy of couch position relative to the radiological focus	Film QA Phantom Film Results	Systematic
Couch stability	Stability of couch performance over time	Daily Focus Precision Test	Systematic
CBCT localization	Accuracy of CBCT definition of radiological focus point	Film QA Phantom Localization	Systematic
CBCT stereotactic space, registration	Accuracy of CBCT definition of the treatment area as well as registration of offset CBCT images	Film QA Phantom Localization Tests	Systematic
CBCT stability	Stability of CBCT performance over time	Daily CBCT Localization Test	Systematic
CBCT to MRI registration	Offset of MRI anatomy compared to CBCT anatomy	Patient Images (TRE, TG‐132)	Systematic
HDMM accuracy	Accuracy of HDMM readout	In‐House Phantom Tests	Systematic
Mask immobilization	Rigidity of mask on stationary phantom	HDMM Log File Analysis	Systematic
Residual corrections post‐CBCT	Remaining HDMM error after CBCT corrections applied	HDMM Log File Analysis	Residual/ Random
Motion during treatment	Patient movement during treatment delivery	HDMM Log File Analysis	Residual/ Random

## 2 METHODS

### 2.1 Base delivery device

**FIGURE 2:**
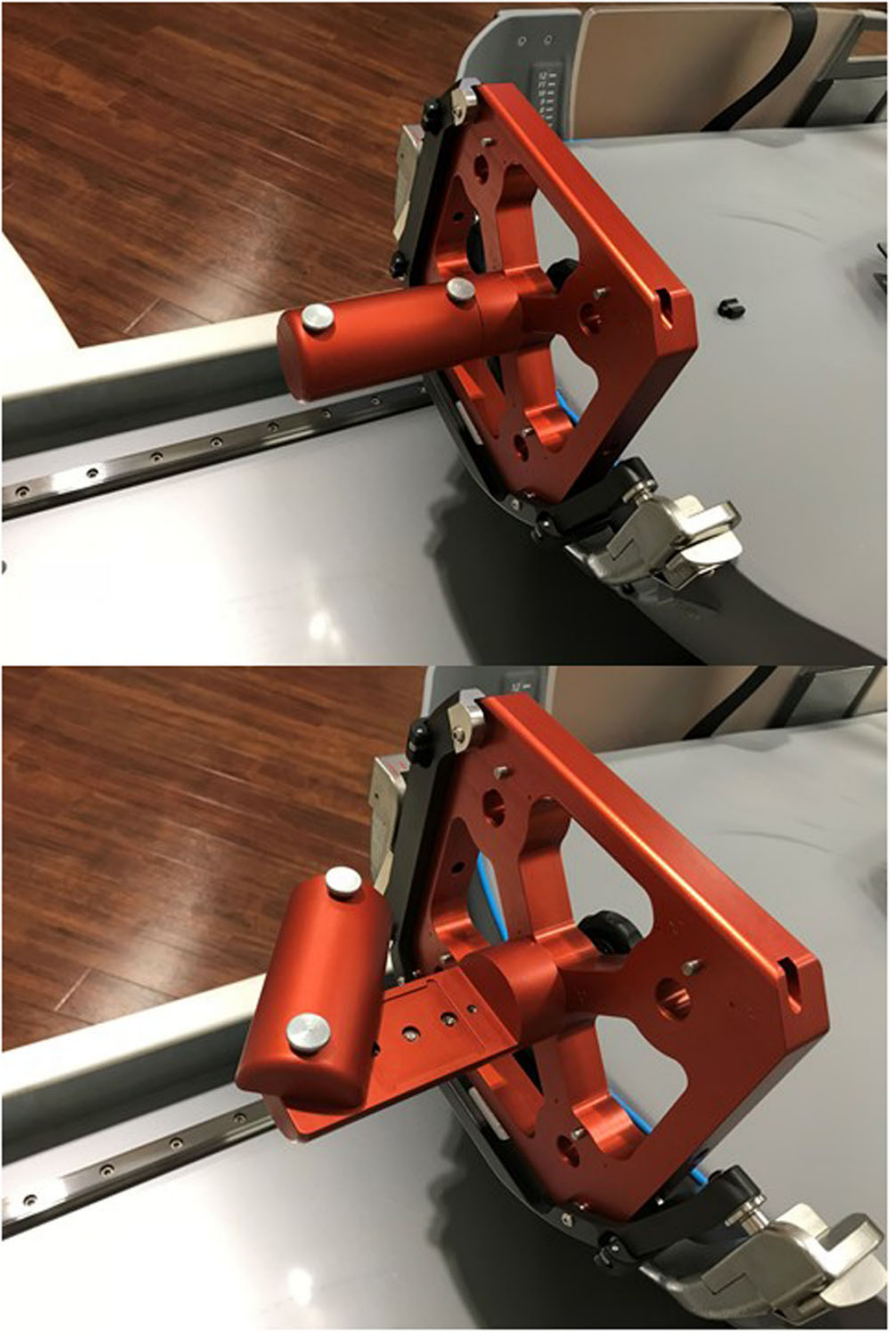
(Top) The Elekta Film phantom in place at reference position to measure x‐z. (Bottom) The Elekta Film phantom open showing the film insert position and the pinprick tool

To determine total workflow uncertainty, each part of the treatment delivery workflow was evaluated with special attention, perhaps, to the novel portions of the workflow. The base uncertainty, or non‐unique portions, of the delivery have been previously studied in the literature and those results were validated here utilizing the Elekta prescribed acceptance tests and trending of the daily QA for those tests:

1. The Elekta‐provided film phantom (shown in Figure 2) was set up on the patient positioning system and a custom cut piece of radiochromic film (EBT3, Ashland Advanced Materials) was placed at the center of the film phantom and pinpricked. The film was then irradiated at a stereotactic center for 2 min with the 4 mm collimator. This was repeated with the phantom rotated to include both axes (x‐z and y‐z) since the film is 2‐dimensional and then with the phantom offset to the different corners of stereotactic space for both phantom orientations at each position. These films were then scanned into FilmQAPro 2016 by an Epson 10000XL transmission scanner and the profiles (radiological center) were analyzed in comparison to the location of the pinprick (mechanical center).
2. The Elekta‐provided and calibrated QA diode tool was used prior to treatments for the daily “focus precision test” for over a year to measure the position of the radiological focus compared to the position calibrated into the patient positioning system. These results were recorded and trended over that time, giving an indication of the stability of this agreement consistent with a recommended methodology involving statistical process control.^32^

### 2.2 Use of MRI for treatment planning

Since the treatment planning is performed based on MRI imaging, the inherent distortion within those images represents another source of systematic uncertainty and is not specific to the frameless technique. The distortion of our 1.5 T Siemens MRI scanner (MAGNETOM Aera, RT Pro Edition) has been assessed in another study and this data will be used for the uncertainty level of our MRI scanner. The 1 × 1 × 1 mm^3^ T1 MPRAGE axial MRI was taken of a cubical grid phantom with known dimensions and 8–14 points in each image plane (coronal, sagittal, and axial) was quantitatively analyzed by vendor software to determine image position versus actual dimensions.^33^ This type B uncertainty data was included in the total uncertainty evaluation.

### 2.3 CBCT localization

Others have already contributed to the knowledge of CBCT's agreement with stereotactic space defined by the traditional frame as well as the long‐term stability of its performance.^34^ This study both confirmed and added to that information by quantifying the ability of the CBCT to accurately identify changes in patient position and then apply corrections to the positioning system.

1. First, onsite data regarding daily CBCT reconstruction of the Elekta QA diode tool with four reference BBs were collected and analyzed. This was done in accordance with Elekta procedures and per TG‐178 recommendations.^7^
2. Second, again Elekta film phantom was used. This phantom can be positioned at the center but also offset towards each corner of stereotactic space at a known distance of 6 cm in both x and y directions. The initial reference CBCT was acquired at the center whereby follow‐up scans were taken with the film insert at the center, rotated 90°, and offset towards the different corners and the corrections recommended by the software will be compared to the known offset distance.
3. Additionally, the placement of film, as for mechanical vs. radiological, was repeated at stereotactic center with the phantom in both orientations (x‐z and y‐z) except that a CBCT of the phantom was acquired and used via treatment planning to define the shot position at the phantom focal point. This shot position was then compared to the known expectation of 100, 100, 100 x, y, z.
4. In addition to the use of CBCT to define stereotactic space, the conversion of this coordinate system to that of the treatment plan was evaluated by testing the co‐registration of MRI with CBCT utilizing the AAPM recommendations from TG Report 132 on QA for image fusion algorithms and software. Target registration error (TRE), defined in the aforementioned report, was used to evaluate the reliability of MRI registrations and by default the quality of stereotactic coordinate transfer to the treatment planning workspace via CBCT. TRE involves the comparison of the coordinates of matching anatomic points in each study after registration.^35^ Three anatomic points were evaluated for 10 different frameless patients for a total of 30 registration points.

### 2.4 HDMM monitoring

To assess the ability of the HDMM camera, a micrometer‐driven phantom was used. The body of which was driven by micrometers in all three translational directions with a resolution of 0.01 mm at a time with a fiducial marker placed in view of the HDMM camera. The HDMM camera establishes a reference position after CBCT acquisition and then monitors for a change from that reference position. The ball‐bearing phantom was set up with a fiducial marker between the HDMM's reference fiducial markers. The phantom was then adjusted with known distances (<1.5 mm) using the micrometer dials in each direction and then the readout of the HDMM will be recorded. This test was repeated in part on a monthly basis over time.

### 2.5 Mask immobilization

To assess the rigidity and reliability of the mask set up, a few different approaches were utilized. An anthropomorphic phantom from Imaging and Radiation Oncology Core (IROC), which is made of thermoplastic material in the shape of a head of standard dimensions, was immobilized in the same fashion as for actual patients. A moisture‐activated head cushion was placed in the adaptor for positioning and then molded to the shape of the back of the head and neck phantom. Then a mask was heated to just under 74°C for 5 min and then removed and attached to the adaptor over the face of the phantom with molding to the different contours of the face. After the mask and head cushion sat for more than the Elekta recommended 7 min, an initial reference CBCT of the phantom was taken with the phantom immobilized on the treatment couch. The mask, head cushion, and phantom were removed from the adaptor and then replaced for repeat imaging and comparison to reference to determine best‐case inter‐fraction reproducibility. The treatment control system discerned and applied corrections both translationally and rotationally to prescribed shot positions to create a “residual” setup error.

Further, the rigidity of the mask was quantified after letting the mask and head cushion sit for a day, re‐setting up the phantom in treatment position with monitoring by the HDMM. The mask immobilization of a phantom was considered the best case scenario since a phantom, though anthropomorphic in design, is stationary and not moving without any human intervention. Additionally, treatment was delivered to a stationary phantom immobilized in the mask while being monitored with the HDMM camera. The readout of the HDMM in this scenario was used to establish a baseline of minimum movement and mask immobilization ability expected during couch motion. A baseline of the systematic and some limited random uncertainty was established by treating this anthropomorphic phantom from the IROC, previously RPC, at MD Anderson Cancer Center clinically as if it were for a real patient.^36^

### 2.6 Random uncertainty – patient HDMM and CBCT data

The above methods will be considered as the primary quantification of systematic uncertainty. To address random uncertainty of the workflow, actual patient treatments must be observed. The performance of each of the Icon‐specific approaches (CBCT, mask immobilization, and HDMM), was evaluated from patient to patient. Gamma Knife delivery log files were obtained and parsed for HDMM marker position over time for each patient using a Python3 script. These log files record the marker position each time the radial position changes from baseline by more than 0.2 mm. The x, y, and z marker coordinates can also be extracted and compared to the post‐CBCT reference position, but it should be noted that x, y, and z are instantaneously recorded for the last window of a 500 ms timeframe whereas the markers radial position is averaged over that time for its record in the log files. For this reason, the x, y, and z coordinates do not agree with the radial displacement as they are an instantaneous readout and do not represent the average value over the last 500 ms. Once this data was extracted, the area under the curve (AUC) analysis was used to calculate the mean position over the treatment relative to the last reference marker position after the most recent CBCT correction. Only marker data during treatment delivery was included in the analysis and this data was truncated at the utilized gating threshold since delivery was paused when the marker was beyond this point, and it would only resume after correction or marker movement back within tolerance.

CBCT imaging and registration that was necessarily repeated during patient treatments were recorded with translationally and rotationally applied corrections from baseline reference CBCT. After CBCT correction, the residual setup error post CBCT was also recorded by the control system in the aforementioned log files. This HDMM marker data were retrospectively collected and analyzed. The patient‐specific random uncertainty related to the HDMM data was analyzed versus various patient‐related factors with the statistical analysis to determine potential predictive variables.^32^

## 3 RESULTS

Measured values are reported below in the format of mean ± standard deviation. In addition, the 95% CI is presented for each uncertainty value.

### 3.1 Base delivery device uncertainty

The results of the film measurements with the Gamma Knife patient positioning system versus radiological focus showed mean values of the uncertainty of –0.04 ± 0.08, 0.01 ± 0.11, and –0.09 ± 0.13 mm in the x‐, y‐, and z‐ directions, respectively. Computation of the 3D vector in quadrature resulted in a mean of 0.21 ± 0.04 mm. The 95% CIs for x, y, z, and total are [–0.09, 0.01], [–0.06, 0.08], [–0.15, –0.03], and [0.19, 0.24], respectively. The results are included in Table 2.

**TABLE 2:** Summary of the uncertainty of the different portions of treatment planning and delivery workflow for frameless Gamma Knife radiosurgery

Mean (1SD)					
Source of uncertainty	*x* (mm)	*y* (mm)	*z* (mm)	Total vector mean (mm)	Error type
MRI Distortion^a^	0.22 (0.04)	0.33 (0.12)	0.44 (0.15	0.63 (0.18)	Systematic
PPS (*n* = 9)	–0.04 (0.08)	0.01 (0.11)	–0.09 (0.13)	0.21 (0.04)	Systematic
PPS Stability (*n* = 116)	0.05 (0.05)	0.00 (0.00)	0.00 (0.00)	0.05 (0.05)	Systematic
CBCT Localization at Center (*n* = 3)	–0.07 (0.03)	0.02 (0.17)	–0.10 (0.17)	0.29 (0.06)	Systematic
CBCT Stereotactic Space (*n* = 18)	0.01 (0.08)	–0.01 (0.083)	0.01 (0.03)	0.10 (0.05)	Systematic
CBCT Stability (*n* = 118)	NA	NA	NA	0.09 (0.03)	Systematic
MRI‐CBCT Registration (*n* = 30)	0.01 (0.40)	–0.07 (0.39)	–0.09 (0.35)	0.62 (0.23)	Systematic
HDMM Accuracy (*n* = 36)	0.01 (0.02)	–0.00 (0.03)	0.02 (0.03)	0.04 (0.04)	Systematic
Mask Immobilization (*n* = 2)	–0.06 (0.03)	0.02 (0.03)	0.09 (0.02)	0.22 (0.10)^c^	Systematic
Post‐CBCT Residual (*n* = 30)	–0.02 (0.17)	–0.02 (0.21)	0.11 (0.37)	0.45 (0.32)^c^	Random/Residual
Motion During Treatment (*n* = 30)	–0.00 (0.28)	–0.06 (0.24)	0.24 (0.27)	0.72 (0.24)^c^	Random/Residual
**Total systematic**	0.25 (0.43)	0.34 (0.47)	0.48 (0.44)	0.99 (0.64)	Systematic
**Total random**	0.02 (0.33)	0.06 (0.32)	0.26 (0.45)	0.85 (0.40)	Random
**Total uncertainty**	0.25 (0.54)	0.34 (0.57)	0.54 (0.64)	1.30 (0.51)	Total Combined

^a^ Data from UMMC institutional study.

^b^ Note that the Total Vector Mean is the averaged quadrature of vector error for each measurement not the quadrature of the x, y, z error for the group.

^c^ Note the difference for the recorded x, y, z, and radial for HDMM logs as discussed in the text.

The consistency of this agreement measured by the QA diode tool showed only deviations in the x‐direction over 116 measurements with a mean of 0.05 ± 0.05 mm with 95% CI [0.05, 0.05], though its readout is only to the tenth of a millimeter perhaps explaining the lack of deviations observed in the y‐ and z‐directions, but still demonstrating great stability over time. These results are also included in Table 2.

### 3.2 CBCT localization

After 118 measurements utilizing the QA diode tool, a mean radial max displacement over time was 0.09 ± 0.03 mm with 95% CI [0.08, 0.09]. The readout is only radially and not shown for each individual cartesian coordinate direction. These results are also included in Table 2.

The film measurements repeated with the definition of stereotactic center utilizing CBCT demonstrated values of the uncertainty of –0.07 ± 0.03, 0.02 ± 0.17, and –0.10 ± 0.17 mm in the x‐, y‐, and z‐directions, respectively. The calculation of the 3D vector of uncertainty was 0.29 ± 0.06 mm. The 95% CIs for x, y, z, and 3D total are [–0.10, –0.03], [–0.17, 0.21], [–0.23, 0.04], and [0.22, 0.35], respectively This data have also been included in Table 2.

Results of the CBCT‐to‐CBCT registration of the film phantom throughout stereotactic space were means of 0.01 ± 0.08, ‐0.01 ± 0.08, and 0.01 ± 0.03 mm for the x‐, y‐, and z‐directions, respectively. The total 3D vector results are 0.016 ± 0.12 mm. The 95% CIs for x, y, z, and 3D total are [–0.02, 0.05], [–0.04, 0.03], [–0.01, 0.02], and [0.09, 0.12], respectively. This data are also shown in Table 2.

Results of the CBCT to MRI registration TRE analysis were means of 0.13 ± 0.4, ‐0.07 ± 0.39, and ‐0.09 ± 0.35 mm for the x‐, y‐, and z‐directions, respectively. The total 3D vector results are 0.62 ± 0.23 mm. The 95% CIs for x, y, z, and 3D total are [‐0.14, 0.17], [‐0.25, 0.10], [‐0.24, 0.07], and [0.54, 0.71], respectively. This data are shown in Table 2, as well.

### 3.3 Mask immobilization

The IROC phantom tracking data during treatment delivery revealed small, registered movements, but larger than the measurement uncertainty of the HDMM system itself. Pre‐treatment CBCT corrections resulted in residual HDMM displacement of –0.09, 0.06, 0.06, and 0.09 mm in the x, y, z, and radial directions, respectively. Throughout the treatment of the phantom, the HDMM tracking showed average HDMM displacement of –0.06 ± 0.03, 0.02 ± 0.03, 0.09 ± 0.02, and 0.22 ± 0.10 mm for x, y, z, and radial, respectively. These results are also included in Table 2.

### 3.4 HDMM system

After 36 measurements, the mean accuracy of the HDMM system was determined to be 0.01 ± 0.02, –0.00 ± 0.03, and 0.02 ± 0.03 mm for x‐, y‐, and z‐directions, respectively. The total 3D vector was 0.04 ± 0.04 mm. The 95% CIs for x, y, z, and 3D total are [0, 0.01], [–0.01, 0.01], [0.01, 0.03], and [0.03, 0.05], respectively. These results are also included in Table 2.

Thirty different patients underwent HDMM monitoring for treatment times between 6.4 and 107.8 min. 16 of the patients received treatment in a single fraction while 14 underwent fractionated regimens of 3–5 treatments for total dose delivery. The average AUC during treatment for all patients was –0.00 ± 0.28, ‐0.06 ± 0.24, 0.24 ± 0.27, and 0.72 ± 0.24 mm for x, y, z, and radial, respectively. The 95% CIs for x, y, z, and 3D total are [–0.07, 0.07], [–0.11, 0], [0.17, 0.30], and [0.63, 0.80], respectively. Also noted was the residual HDMM motion position post CBCT correction which was averaged to be –0.02 ± 0.17, ‐0.02 ± 0.21, 0.11 ± 0.37, and 0.45 ± 0.32 mm for x, y, z, and radial, respectively. The 95% CIs for x, y, z, and 3D total are [–0.06, 0.02], [–0.08, 0.03], [0, 0.23], and [0.34, 0.57], respectively. These results are also in Table 2. Note that there is a discrepancy between the quadrature of the recorded x, y, and z values and the recorded radial displacement. This is because of the way that the data is recorded in the log files which is discussed above in the methods section. The radial displacement is seen as a better measure of the patient's overall displacement recorded by the HDMM.

Unfortunately, the HDMM only reads the position of the nose fiducial marker. The nose can move independently of the skull, though the averaging of the marker position helps with this in some regard. However, even assuming the nose as stationary, it may not always be completely correlative of the translations of different portions of the brain as though the nose marker may move due to head translation or rotation, the HDMM will only ever discern translation of this nose marker. Wright et al. have investigated the validity of using this technique to track intracranial targets. They concluded that much of the time patient anatomy was generally displaced roughly half the value of the nose marker, but there were instances where anatomy could be displaced to a higher degree than the marker such as when the cranium is rotated around the y axis (anterior/posterior).^37^ This presents a challenge in translating this marker displacement to actual uncertainty related to intracranial targets, so perhaps semi‐conservatively, but not so much in some cases, the uncertainty of the target position is being considered equal to the nose marker displacement for the purposes of this study.

### 3.5 Total workflow uncertainty

The different areas of uncertainty have been added in quadrature based on the recommendations of the International Commission on Radiation Units and Measurements.^38^ The components were assumed to not be correlated and to have normal distributions when summing. This methodology is considered reasonable and effective when compiling many and various sources of error and has been demonstrated by several groups.^14^, ^18^, ^26^, ^27^, ^39^ The summary of the total workflow uncertainty can be seen in Table 2 including one standard deviation of the mean for each component. The text above also includes the 95% CI. It should be noted that the values in the x, y, and z directions represent means calculated from positive and negative values while the radial mean is a vector and is calculated from values always representing the absolute offset or the vector offset. Additionally, one should again note the discrepancy in x, y, z, and radial for the HDMM log files as mentioned above.

## 4 DISCUSSION

Upon this total workflow uncertainty assessment, the assumption of frameless Gamma Knife uncertainty to be less than 1 mm may not be valid in many cases. Supposing the measured uncertainty exhibits a normal distribution around the mean of 1.30 mm and with the measured standard deviation of 0.51 mm, it can be estimated that there is about a 27.5% probability that the mean total vector uncertainty would be 1.00 mm or less for a given patient. This study appears to warrant consideration of setup margin during the simulation and treatment planning of these patients. Of course, setup margin should be considered on a case‐by‐case basis as it depends on whether the risk of patient injury due to tumor progression as a result of underdosing outweighs the risk of patient harm due to normal tissue injury such as radionecrosis. Similar logic is shared by our HYTEC colleagues in the choice of applied normal tissue goals.^40^ Rationally, the setup margin could be applied to the minimum level needed in each cartesian direction, rather than a global 1 mm, since the patient‐specific uncertainty varied around the mean of 0 for many of the uncertainty components, thought setup margin application directly within GammaPlan is limited in the current software version. It should also be noted that much of this data is specific to our institution that of course indicates that the uncertainty may be more or less at another clinic, especially since clinicians may become more practiced over time in patient management and immobilization which appears to be the larger side of the uncertainty. Others corroborate the data presented in this work to an extent, but it has also been shown that adding errors in quadrature is not always the best estimate for actual patient experienced uncertainty. Therefore, this data can be viewed as an important, but also a conservative estimate of the uncertainty of a procedure due to the limited discernability in the overlap between different sources of error and how they are added together.^2^, ^25^, ^41^

Indeed, the prospect of setup margin is not as cut and dry as one might suggest since setup margin, by nature, includes more normal tissue. However, missing the target also means severe repercussions in either toxicity due to disease progression or lack of treatment effectiveness. The impact of the uncertainty in frameless Icon treatment also depends on the target where targets such as trigeminal neuralgia may be more sensitive. In such cases, the choice of the frame‐based technique is also an option to reduce uncertainty rather than compensate with setup margin. Practically this occurs within our own clinic as physicians often choose frame immobilization based on the target size and/or type. Uncertainty also may be minimized to some extent. Some data have indicated patient factors related to higher movement within the Icon mask immobilization and therefore these factors may be considered when choosing a treatment technique. Further improvements in MRI imaging distortion or image fusion techniques would also lead to significant uncertainty reduction.

## 5 CONCLUSIONS

The total uncertainty of the frameless Icon procedure has been quantitatively evaluated. Based on that evaluation, application of some setup margin may be recommended, but any application of setup margin, especially in radiosurgery, should be carefully considered, perhaps even on a patient‐specific basis. If desired, this data could be applied to setup margin using various recipes that have been described in the literature.^26^, ^27^, ^42^

## CONFLICT OF INTEREST

The authors declare that they have no conflict of interest.

## AUTHOR CONTRIBUTIONS

William Duggar – Conception, literature review, data collection, project design, manuscript writing, and manuscript review

Bart Morris – Data collection and manuscript review

Rui He – Data collection and manuscript review

Claus Yang – Data Collection, project design, and manuscript review
